# Predictive Features of Severe Acquired ADAMTS13 Deficiency in Idiopathic Thrombotic Microangiopathies: The French TMA Reference Center Experience

**DOI:** 10.1371/journal.pone.0010208

**Published:** 2010-04-23

**Authors:** Paul Coppo, Michael Schwarzinger, Marc Buffet, Alain Wynckel, Karine Clabault, Claire Presne, Pascale Poullin, Sandrine Malot, Philippe Vanhille, Elie Azoulay, Lionel Galicier, Virginie Lemiale, Jean-Paul Mira, Christophe Ridel, Eric Rondeau, Jacques Pourrat, Stéphane Girault, Dominique Bordessoule, Samir Saheb, Michel Ramakers, Mohamed Hamidou, Jean-Paul Vernant, Bertrand Guidet, Martine Wolf, Agnès Veyradier, for the French Reference Center for Thrombotic Microangiopathies

**Affiliations:** 1 Service d'Hématologie et de Thérapie Cellulaire, AP-HP, Hôpital Saint-Antoine, Paris, France; 2 UPMC Univ Paris 06, Paris, France; 3 INSERM UMR_S 707, and UPMC, Univ Paris 06, Paris, France; 4 Service de Néphrologie, Hôpital Maison Blanche, Reims, France; 5 Service de Réanimation Médicale, Hôpital Charles Nicolle, Rouen, France; 6 Service de Néphrologie - Médecine Interne, Hôpital Sud, Amiens, France; 7 Service d'Hémaphérèse, Hôpital de la Conception, Marseille, France; 8 Service de Néphrologie, Centre Hospitalier de Valenciennes, Valenciennes, France; 9 Service de Réanimation Polyvalente, AP-HP, Hôpital Saint-Louis, Université Paris 7 Denis Diderot, Paris, France; 10 Service d'Immunopathologie, AP-HP, Hôpital Saint-Louis, Université Paris 7 Denis Diderot, Paris, France; 11 Service de Réanimation Polyvalente, AP-HP, Hôpital Cochin, Université Paris 5, Paris, France; 12 Service de Néphrologie, AP-HP, Hôpital Tenon, UPMC Univ Paris 6, Paris, France; 13 Service de Néphrologie et Immunologie Clinique, CHU Rangueil, Toulouse, France; 14 Service d'Hématologie Clinique et de Thérapie Cellulaire, CHU Dupuytren, Limoges, France; 15 Service d'Hématologie Clinique et de Thérapie Cellulaire, AP-HP, Hôpital de la Pitié-Salpêtrière, UPMC Univ Paris 6, Paris, France; 16 Service de Réanimation Médicale, Centre Hospitalier Universitaire, Caen, France; 17 Service Médecine Interne A, Hôpital Hôtel-Dieu, Nantes, France; 18 Service de Réanimation Médicale, AP-HP, Hôpital Saint-Antoine, Paris, France; 19 Service d'Hématologie Biologique, AP-HP, Hôpital Antoine Béclère, Clamart, et U770 Inserm, Université Paris-Sud 11, Le Kremlin-Bicêtre, France; University of Giessen Lung Center, Germany

## Abstract

Severe ADAMTS13 deficiency occurs in 13% to 75% of thrombotic microangiopathies (TMA). In this context, the early identification of a severe, antibody-mediated, ADAMTS13 deficiency may allow to start targeted therapies such as B-lymphocytes-depleting monoclonal antibodies. To date, assays exploring ADAMTS13 activity require skill and are limited to only some specialized reference laboratories, given the very low incidence of the disease. To identify clinical features which may allow to predict rapidly an acquired ADAMTS13 deficiency, we performed a cross-sectional analysis of our national registry from 2000 to 2007. The clinical presentation of 160 patients with TMA and acquired ADAMTS13 deficiency was compared with that of 54 patients with detectable ADAMTS13 activity. ADAMTS13 deficiency was associated with more relapses during treatment and with a good renal prognosis. Patients with acquired ADAMTS13 deficiency had platelet count <30×10^9^/L (adjusted odds ratio [OR] 9.1, 95% confidence interval [CI] 3.4–24.2, *P*<.001), serum creatinine level ≤200 µmol/L (OR 23.4, 95% CI 8.8–62.5, *P*<.001), and detectable antinuclear antibodies (OR 2.8, 95% CI 1.0–8.0, *P*<.05). When at least 1 criteria was met, patients with a severe acquired ADAMTS13 deficiency were identified with positive predictive value of 85%, negative predictive value of 93.3%, sensitivity of 98.8%, and specificity of 48.1%. Our criteria should be useful to identify rapidly newly diagnosed patients with an acquired ADAMTS13 deficiency to better tailor treatment for different pathophysiological groups.

## Introduction

Thrombotic microangiopathies (TMA) represent a rare and heterogeneous group of diseases defined by microangiopathic hemolytic anemia with peripheral thrombocytopenia and organ failure of variable severity. TMA encompass thrombotic thrombocytopenic purpura (TTP), typically characterized by central nervous system (CNS) involvement, and hemolytic uremic syndrome (HUS) in which severe renal involvement is the prominent abnormality. TMA may also be associated with various conditions such as pregnancy, human immunodeficiency virus (HIV) infection, cancer and chemotherapy, transplantation or medications.

TTP results from excessive platelet aggregation in multiple organs with, consequently, a dramatical increase in shear stress caused by the accumulation of unfolded high-molecular weight von Willebrand factor multimers in plasma. Failure to process these multimers into smaller, less adhesive forms is related to a dysfunction in ADAMTS13, an enzyme specifically involved in this process (Figure S1). ADAMTS13 deficiency may result from mutations of the encoding gene or from autoantibodies in the acquired form [1]. Autoantibodies may alter enzyme function through two nonexclusive mechanisms. The first mechanism is a direct neutralizing effect, as evidenced by functional in vitro assays in which plasma from patients with TTP inhibits ADAMTS13 activity in normal human plasma. The second mechanism is an opsonization process, involving the formation of immune complexes with ADAMTS13, which are subsequently cleared by phagocytes [2], [3]. Severe ADAMTS13 deficiency was reported in 33% to 90% of patients with TTP, whereas ADAMTS13 activity was found usually normal or slightly reduced in patients with HUS or other causes of thrombocytopenia [4]–[10]. ADAMTS13 deficiency on diagnosis was reported to be associated with a better survival, though in those studies patients with a detectable ADAMTS13 activity usually had confounding factors such as associated severe conditions involved in cytopenias and organ injury, which precludes definitive conclusions [10].

The involvement of ADAMTS13 in TTP pathophysiology opens the promising perspective of targeted therapies in association with the current, plasma-based, standard therapy. In particular, these include immunomodulatory drugs that allow depleting autoreactive B lymphocytes in patients with acquired, antibody-mediated ADAMTS13 deficiency. In that regard, the humanized anti-CD20 monoclonal antibody rituximab produced high levels of response in patients with refractory or relapsing acquired TTP [11]–[13] as a second line treatment. In contrast, the efficiency of rituximab in TMA with a detectable ADAMTS13 activity remains uncertain. Thus, future therapeutic assays involving immunomodulation as a first line treatment will reasonably be targeted first to patients with an autoantibody-mediated severe ADAMTS13 deficiency. In this regard, the early administration of rituximab in acquired severe ADAMTS13 deficiency-associated TTP recently showed encouraging results by limiting treatment duration in slow responders with a non optimal response to standard plasma exchange (PE)-based treatment through a faster and durable increase in ADAMTS13 activity ([14] and manuscript submitted).

Therefore, the rapid recognition of a severe acquired ADAMTS13 deficiency is necessary 1) to facilitate the early diagnosis of TTP and 2) to identify a subgroup of patients who may be the best target for future assays evaluating the place of immunomodulators in the therapeutic scheme. Today, many methods are available to measure ADAMTS13 activity as a first line test and anti-ADAMTS13 IgG as a second line test if ADAMTS13 activity id found markedly decreased [1]. Among these methods, the one using the FRETS-VWF73 substrate [15] combines both rapidity and reliability. However, until now, all ADAMTS13-related assays still belong to the field of medical research and have not been validated as routine tests. For this main reason and also for cost issues, the ADAMTS13-related assays are not accessible to any laboratory and remain limited to specialized expert laboratories. Furthermore, considering the low incidence of TMA and especially TTP [10], the national plans for rare diseases organized in various countries recommend that specialized analysis dedicated to these pathologies remain centralized in a very limited number of university hospital laboratories. As ADAMTS13 activity measurement is crucial to document in any patient with a TMA suspicion, that means that some hospitals will not be able to beneficiate of the ADAMTS13 assay locally in emergency although this would facilitate the early diagnosis and management of TTP.

We and others previously reported from a preliminary retrospective study that idiopathic autoimmune TTP with a documented severe acquired ADAMTS13 deficiency was characterized by more severe thrombocytopenia, mild renal involvement [16,17 and more recently 18], and various immunopathological features [16], [19]. However, whether specific clinical and laboratory manifestations may be used to predict accurately and rapidly ADAMTS13 dysfunction has not been evaluated so far. Thus, the primary objective of this study was to establish a score to predict ADAMTS13 severe deficiency using standard clinical and biological parameters. In that regard, we performed a cross-sectional analysis of our national Registry from 2000 to 2007 allowing the analysis of 214 patients with idiopathic TMA. As a secondary objective of this study, we focused on both initial clinical presentation and short term outcome as a function of ADAMTS13 activity to evaluate the relevance of a pathophysiology-based classification of TMA to guide future therapeutic assays.

## Methods

### Study Design

The TMA Registry of the French Reference Center for the management of thrombotic microangiopathies (www.cnr-mat, in preparation) was established in October 2000 to prospectively and systematically collect comprehensive clinical and biological data on patients with various forms of TMA and to create a repository including plasma and mononuclear cells on diagnosis and during follow-up (http://www.orpha.net/consor/cgi-bin/index.php?lng=FR, key-word: PTT or TTP). To manage patients with TMA consistently throughout our country and to have a comprehensive registry, we officially defined at least one leading academic center in each French region (members listed in appendix). All defined centers, as well as their respective affiliated regional centers, are involved in the management of patients with TMA in concert with the reference center, according to consensual recommendations or results of clinical trials (http://asso.orpha.net/ORPHAWEB/cgi-bin/file/Plan_Maladies_Rares.pdf).

In the present work, adult (>18 years old) patients were studied prospectively from October 2000 to October 2007; they were recruited consecutively and nonselectively from intensive care units and departments of hematology, internal medicine and nephrology in 17 leading French centers and their respective affiliated regional centers. Patients treated for an initial diagnosis of idiopathic TMA and in whom an associated condition was diagnosed were subsequently excluded from the current study. Patients with severe (<5% of normal activity) ADAMTS13 deficiency were compared for clinical, biological, and immunopathological features to patients with detectable (≥20%) ADAMTS13 activity. Patients with an ADAMTS13 activity between 5–20% in whom either inhibitory or non-inhibitory anti-ADAMTS13 antibodies were detectable were considered as having a acquired mild ADAMTS13 deficiency and were included in the group of patients with a undetectable ADAMTS13 activity.

### Patients and Treatment

All adult patients who satisfied the following diagnostic criteria for TMA were enrolled in the study. This study was approved by the Saint-Antoine Hospital Ethical committee/Institutional review board (http://www.orpha.net/consor/cgi-bin/ResearchTrials_RegistriesMaterials.php?lng=FR&data_id=30475&Nome%20del%20registro%20di%20materiali=Registre-du-reseau-d-etude-des-microangiopathies-thrombotiques&title=Registre-du-reseau-d-etude-des-microangiopathies-thrombotiques&search=ResearchTrials_RegistriesMaterials_Simple). Written informed consent was obtained from all patients. The criteria included the presence of Coombs-negative microangiopathic hemolytic anemia (schistocytes on peripheral blood smear) and acute thrombocytopenia (<150×10^9^/L). Patients with TMA were excluded if organ failure and cytopenias may have been related to an associated condition and not only to the microangiopathic process, that is, solid organ or hematopoietic stem cell transplant, cancer and chemotherapy, the syndrome of hemolysis, elevated liver enzymes, and low platelet count (HELLP), active systemic lupus erythematosus (SLE) or systemic sclerosis, HIV infection and severe sepsis [16]. Treatment was administered per protocol according to written recommendations detailed in previous studies [20], [21] and irrespective of ADAMTS13 activity and anti-ADAMTS13 antibodies status.

### Analysis

Data were collected on diagnosis for each patient as previously described [16]. All data were collected using an established questionnaire and recorded in a computerized databank.

Blood collection, plasma preparation, and measurement of ADAMTS13 activity were performed as previously described [6]. ADAMTS13 activity was considered undetectable if <5% of normal activity. Inhibitory anti-ADAMTS13 antibodies were sought in patients when ADAMTS13 activity was <20%. Since 2006, anti-ADAMTS13 antibodies were sought by ELISA as previously described [3] when ADAMTS13 activity was <20%. In some patients included before 2006, anti-ADAMTS13 antibodies were sought by ELISA retrospectively from our serum collection. ADAMTS13 deficiency was considered acquired if associated with detectable inhibitor and/or anti-ADAMTS13 antibodies, or if ADAMTS13 deficiency recovered completely or partially (≥20%) after remission. Anti-ADAMTS13 IgG titer was considered positive for values >15 U/mL. In patients with anti-ADAMTS13 IgG titer at the limit of the positivity threshold (15 to 20 U/mL), ADAMTS13 activity was investigated during remission, and the diagnosis of acquired ADAMTS13 deficiency was retained with certainty in patients for whom ADAMTS13 activity recovered completely or partially.

### Definitions

Response to treatment was defined from previous works on the basis of the experience of various National centers, and were in part published elsewhere [21]. A complete response was defined as the disappearance of possible neurological manifestations and complete platelet count recovery (>150×10^9^/L) for at least 2 days and durable remission was a complete response that persisted more than 30 consecutive days from the first day of platelet count recovery (this period of time included the maintenance treatment by TPE). The time to durable remission was defined as the number of days from the first day of treatment to the first day from which platelet count did not worsen for at least 30 consecutive days. Relapse was defined as the reappearance of neurological manifestations and/or a recurrence of thrombocytopenia (<100×10^9^/L at least 2 days) without any other identifiable cause, after durable remission achievement. Flare-up was defined as an exacerbation of the disease characterized by a occurrence/recurrence of neurological manifestations and/or a recurrence of thrombocytopenia (<100×10^9^/L at least 2 days) and/or a worsening of thrombocytopenia without any other identifiable cause, before the achievement of a durable remission.

### Statistical Analysis

Continuous variables were summarized by mean (standard deviation). Wilcoxon rank-sum test was used to compare continuous variables, and chi-square test or Fisher exact test was used to compare binary data. Data were analyzed using SAS 9.1 (SAS Institute, Cary NC).

We developed an algorithm to predict severe ADAMTS13 deficiency at clinical presentation. Variables significantly associated with severe ADAMTS13 deficiency were tested in multivariate logistic regression with stepwise selection [22]. Selected continuous variables were dichotomized with reference to the threshold maximizing the Youden Index (sensitivity plus specificity minus one) and then rounded to the nearest easy-to-recall value. Two predictive binary scores were calculated: 1) a score minimizing the number of false-positive diagnoses (ie, high specificity and positive predictive value) taking the value of 1 when all criteria were positive, and 0 otherwise; 2) a score minimizing the number of false-negative diagnoses (ie, high sensitivity and negative predictive value) taking the value of 1 when at least one criterion was positive, and 0 otherwise.

Internal validation of the algorithm was performed by the bootstrap resampling technique [23]. Five hundred samples were drawn with replacement from the original data set, of the same size and event probability as the original data set. The predictive performance was assessed by the median (95% confidence interval) of the following indicators: 1) area under the receiver operating characteristic curve (AUROC) where AUROC varied between 0.5 and 1.0 (the higher, the better); 2) specificity, sensitivity, and positive and negative predictive values of the two predictive binary scores.

## Results

The Registry included 361 patients with TMA, of which 118 had an associated condition and 243 were idiopathic. Twenty-nine patients were excluded because of insufficient collected data, leaving 214 patients for analysis.

**Figure 1:**
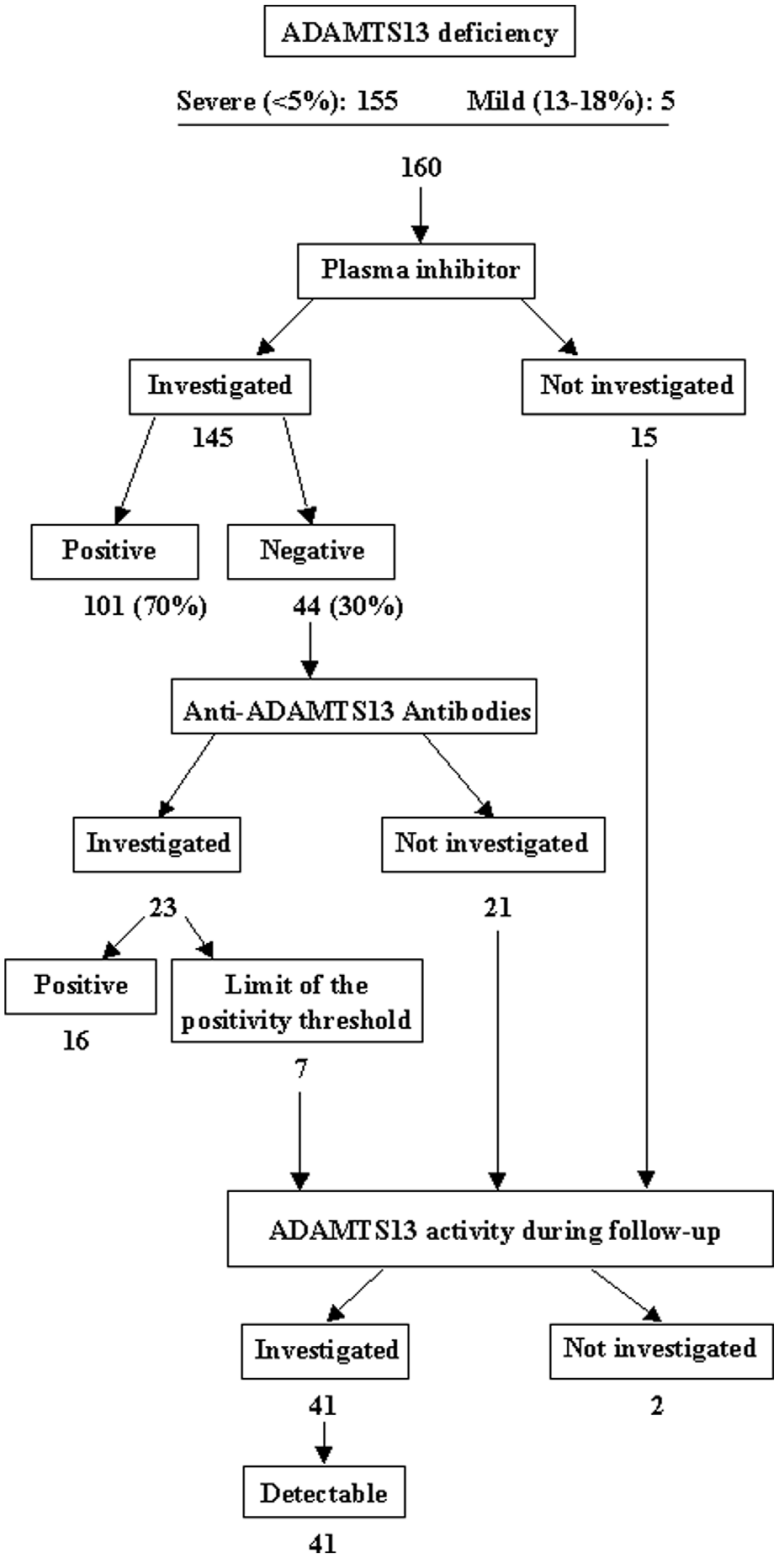
Survey of ADAMTS13 inhibitor and anti-ADAMTS13 antibodies on diagnosis in patients with a severe (<5%) or a mild (13–18%) ADAMTS13 deficiency.

**Figure 2:**
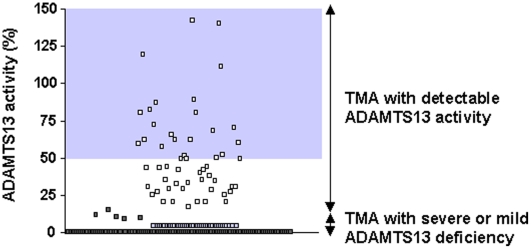
ADAMTS13 value on diagnosis. Dark grey squares: deficient ADAMTS13 activity with inhibitory or non-inhibitory anti-ADAMTS13 antibodies; light grey squares: deficient ADAMTS13 activity with suspected inhibitory or non-inhibitory anti-ADAMTS13 antibodies; white squares: detectable (≥20%) ADAMTS13 activity. Grey area delimits ADAMTS13 values of normal subjects. Abbreviation: TMA, thrombotic microangiopathy.

Severe ADAMTS13 deficiency occurred in 155 patients. Five additional patients had a mild ADAMTS13 deficiency that ranged from 13% to 18% with a detectable plasma inhibitor (1 case) or with anti-ADAMTS13 antibodies (4 cases) as assessed by ELISA. Overall, 160 patients (74.8%) were referred to as the deficient group. ADAMTS13 deficiency was acquired in all investigated cases (Figure 1). In the remaining 54 of 214 patients (25.2%), referred to as the detectable group, the mean ADAMTS13 activity was 51.4 (29)% (Figure 2). Among these patients, anti-ADAMTS13 antibodies were sought in 10 cases (including 4 patients with ADAMTS13 activity ranging from 20% to 35%) and were below the positive threshold level.

Table 1 details the clinical characteristics of the 214 patients according to ADAMTS13 activity. There was no association between ADAMTS13 activity and sex, weight, CNS involvement, autoimmune disease and fever. Patients in the deficiency group were younger (*P*<.001).

**Table 1:** Clinical Characteristics of 214 Patients with Thrombotic Microangiopathy According to ADAMTS13 Activity.

	Deficiency group (*n* = 160)	Detectable group (*n* = 54)	*P* Value
Previous TMA episode	13 (8%)	5 (9%)	.98
Autoimmune disease	27 (17%)^1^	7 (13%)^2^	.64
Family history of autoimmune disease	26 (16%)	10 (19%)	.86
Age, y	39.4 (15.5)	50.6 (19.9)	<.001
Body weight, kg	69.9 (18.4)	66.1 (14.0)	.15
Female	118 (74%)	42 (78%)	.56
Fever	51 (32%)	14 (26%)	.39
Ethnicity^3^			
North Africans	26 (17%)	0 (%)	-
Other Caucasians	116 (75%)	48 (89%)	.01
Afro-Caribbeans	12 (8%)	2 (4%)	-
CNS involvement	94 (59%)	35 (65%)	.43
Headache	37 (23%)	7 (13%)	-
Confusion	29 (18%)	9 (17%)	-
Seizure	11 (7%)	14 (26%)	-
Focal deficiency	38 (24%)	11 (20%)	-
Coma/vigilance disturbance	28 (18%)	17 (31%)	-

Data are presented as mean (standard deviation) or number (percent).

1 Systemic lupus erythematosus (5 cases); idiopathic thrombocytopenic purpura (3 cases); autoimmune endocrinopathies (type 1 diabetes: 2 cases; autoimmune thyroiditis: 1 case); ankylosing spondylitis(2 cases); ulcerative colitis (2 cases), and 1 case each of cutaneous lupus, liver granulomatosis, chronic polyarthritis, psoriasis, juvenile chronic polyarthritis, primary biliary cirrhosis, primary Raynaud phenomenon, eczema, livedo, urticaria, lichen planus, and celiac disease.

2 Autoimmune thyroiditis (2 cases); and 1 case each of rheumatoid arthritis, Crohn disease, psoriasis, type 2 cryoglobulinemia, and Sneddon syndrome.

3 After exclusion of 2 patients of other origin and 8 patients with undisclosed origin.

Abbreviations: CNS, central nervous system; TMA, thrombotic microangiopathy.

Hemoglobin levels were slightly lower (*P* = .06) and reticulocyte count was higher (*P*<.0001) in patients in the deficiency group (Table 2). Levels of LDH were comparable in both groups. Thrombocytopenia was more profound in patients in the deficiency group (*P*<.0001). Serum creatinine level was higher in patients in the detectable group (*P*<.0001), and estimated GFR was lower (*P*<.0001). Patients in the deficiency group were more likely to have detectable ANA (*P*<.001) (Table 2). Antinuclear antibodies (ANA) titers were apparently comparable between both groups (not shown) and ranged from 1∶80 (36 patients) to 1∶5000 (3 patients), through 1∶160 (17 patients), 1∶320 (15 patients), 1∶640 (12 patients), 1∶1280 (11 patients) and 1∶2560 (1 patient). Titers were unavailable for 3 patients.

**Table 2:** Results of Laboratory Testing in 214 Patients with Thrombotic Microangiopathy According to ADAMTS13 Activity.

	Deficiency group (*n* = 160)	Detectable group (*n* = 54)	*P* Value
Hemoglobin level, g/dL	8.1 (2.2)	8.7 (2.1)	.06
Reticulocyte count, ×10^9^/L	185 (118)	106 (80.1)	<.0001
LDH level, U/L	6.0 (4.6)	5.8 (3.5)	.70
Platelet count, ×10^9^/L	17.4 (14.2)	66.6 (49.3)	<.0001
Creatinine level, µmol/L mg/dL	114 (68.4) 1.29 (0.77)	454 (326) 5.13 (3.68)	<.0001
Estimated GFR, mL/min	80.6 (33.3)	35.0 (59.2)	<.0001
ANA	85 (53%)^1^	13 (24%)^2^	<.001
Anti-dsDNA antibodies	9 (7%)^3^	0 (0%)^4^	.21
Anticardiolipin antibodies	14 (11%)^3^	9 (20%)^5^	.11

Data are presented as mean (standard deviation) or number (percent).

1 21 patients had anti-extractable nuclear antigen antibodies, including anti-SSA antibodies in 12 cases, anti-SSA antibodies associated with anti-SSB antibodies in 5 cases, and anti-U1-snRNP antibodies in 4 cases.

2 1 patient had anti-SSA antibodies.

The analysis was performed on 127^3^, 35^4^ and 45^5^ patients.

Abbreviations: ANA, antinuclear antibodies; Anti-dsDNA, anti-double-stranded DNA; GFR, glomerular filtration rate.

Survival, time to durable platelet count recovery, and plasma volumes infused until durable complete remission were comparable between patients in the deficiency and detectable groups (*P* = .73, .36 and .15, respectively; Table 3). However, patients in the detectable group more frequently experienced end-stage renal disease following the TMA episode (*P*<.0001). Steroids and vincristine were used more frequently in patients in the deficiency group (*P* = .001 and *P* = .06, respectively) and splenectomy was performed equally in both groups.

**Table 3.** Treatment and Outcome in 214 Patients with Thrombotic Microangiopathy According to ADAMTS13 Activity.

**Table 3:** Treatment and Outcome in 214 Patients with Thrombotic Microangiopathy According to ADAMTS13 Activity.

	Deficiency group (*n* = 160)	Detectable group (*n* = 54)	*P* Value
Plasma volume, mL/kg^1^	762 (678)	686 (759)	.15
Steroids^2^	130 (81%)	32 (59%)	.001
Rituximab^3^	27 (17%)	0 (0%)	-
Vincristine	35 (22%)	5 (9%)	.06
Splenectomy	7 (4%)	1 (2%)	.66
Time to platelet count recovery, days, median (25^th^–75^th^ percentile)	16 (7–27)	11 (5–38)	.36
Survival	142 (89%)	47 (87%)	.73
Flare-up episode(s)	76 (51%)	13 (26%)	<.01
Relapse^4^	28 (20%)	7 (14%)	.37
ESRD	0 (0%)	10 (21%)	<.0001

Patient Characteristics	Adjusted Odds Ratio	95% CI	*P* Value
Creatinine level ≤200 µmol/L (2.26 mg/dL)	23.4	8.8–62.5	<.001
Platelet count ≤30×10^9^/L	9.1	3.4–24.2	<.001
Positive ANA	2.8	1.0–8.0	<.05

Data are presented as mean (standard deviation) or number (percent).

1 Plasma volume refers to plasma volume infused until durable complete remission.

2 Posology was 1 to 1.5 mg/kg/day for 3 weeks, with a subsequent progressive decrease within the following weeks.

3 Four 375 mg/m2 infusions were performed within 2 to 3 weeks immediately after a PE session.

4 The incidence rate (%) was calculated by dividing the number of patients who relapsed by the number of survivors.

Abbreviation: ESRD, end-stage renal disease.

In the deficiency group, 27 patients received 2 infusions (4 cases), 3 infusions (2 cases), or 4 infusions (21 cases) of rituximab for refractory disease (8 cases), flare-up (11 cases), or chronic relapsing disease with persistent acquired severe ADAMTS13 deficiency (8 cases). Two patients died at 17.5 (3.5) days despite 2 salvage infusions of rituximab. The remaining patients recovered platelet count after 25.1 (15) days. Five patients experienced a relapse 1 (0.72) year following diagnosis, of favorable outcome with PE and rituximab. One patient experienced 2 additional relapses 18 and 30 months later, of favorable outcome again with rituximab and PE. Two patients with a persistent severe ADAMTS13 deficiency after remission achievement were treated with pre-emptive infusions of rituximab, allowing a complete recovery of ADAMTS13 activity. In both cases however, ADAMTS13 recovery was transient, which required additional pre-emptive infusions of rituximab (every ∼18 months for 3 and 5 years, respectively). In the 2 remaining patients, ADAMTS13 activity is persistently normal after 2 and 3 years of follow-up, respectively.

The incidence of relapse did not clearly differ between both groups. However, episodes of flare-up were more frequently observed in patients in the deficiency group (*P*<.01).

Overall mean follow-up was 17.8 (24.2) months.

Three variables were selected in multivariate logistic regression to predict severe ADAMTS13 deficiency at clinical presentation (Table 4): creatinine level, platelet count, and presence of ANA. The AUROC decreased slightly from 0.951 to 0.930 when continuous variables were dichotomized at thresholds of 200 µmol/L for creatinine level and 30×10^9^/L for platelet count. Internal validation by bootstrap resampling showed a high level of accurate prediction of the model (median AUROC, 0.911; 95% CI, 0.868-0.949). When all three criteria were present (creatinine level ≤200 µmol/L, platelet count ≤30×10^9^/L, and positive ANA), specificity was 98.1% (95% CI, 94.4–100%), and positive predictive value was 98.7% (95% CI, 96.4–100%), minimizing the number of false-positive diagnoses. When at least one criterion was present (creatinine level ≤200 µmol/L, platelet count ≤30×10^9^/L, or positive ANA), sensitivity was 98.8% (95% CI, 96.9–100%), and negative predictive value was 93.3% (95% CI, 85.2–100%), minimizing the number of false-negative diagnoses (Table 5).

**Table 4:** Association Between Patient Characteristics and ADAMTS13 Deficiency Using Multivariate Analysis.

	At Least 1 Positive Criterion	All 3 Criteria Positive
Sensitivity	98.8 (96.9–100)	46.9 (41.3–53.1)
Specificity	48.1 (38.9–59.3)	98.1 (94.4–100)
Positive predictive value	85.0 (82.6–87.7)	98.7 (96.4–100)
Negative predictive value	93.3 (85.2–100)	38.6 (35.8–41.9)

Abbreviations: ANA, antinuclear antibodies; CI, confidence interval by bootstrap resampling technique.

**Table 5:**
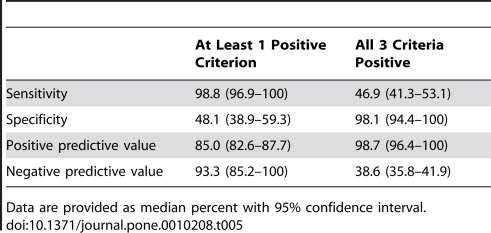
Internal Validation to Predict Severe ADAMTS13 Deficiency at Clinical Presentation.

Data are provided as median percent with 95% confidence interval.

In the original data set, only one patient was classified as a false positive using all three criteria (ie, detectable ADAMTS13 activity despite positive ANA, platelet count ≤30×10^9^/L, and creatinine level ≤200 µmol/L), and results of testing for anti-ADAMTS13 antibodies by ELISA were negative. In contrast, 2 patients were classified as false negative using all three criteria (ie, severe ADAMTS13 deficiency despite negative ANA, platelet count >30×10^9^/L and creatinine level >200 µmol/L). Serum creatinine level ≤200 µmol/L and platelet count ≤30×10^9^/L had the stronger association with a severe ADAMTS13 deficiency (Adjusted Odds ratio >20 and 9, respectively). By using those 2 criteria, we had a very low number of misclassified patients. Indeed, 6 patients with detectable ADAMTS13 activity were classified as a false-positive diagnosis of ADAMTS13 deficiency. In addition, 3 patients with a deficient ADAMTS13 activity were classified as false-negative diagnoses of ADAMTS13 deficiency.

## Discussion

TMA are rare diseases and only registries allowing the analysis of several hundreds of patients may be the basis of reliable data about pathophysiologic, diagnostic and therapeutic issues. The strengths of our study include consistency in evaluation and management (that is, we included patients within a limited period of time, investigated them in the same manner according to consensual national recommendations, determined ADAMTS13 activity in a central laboratory, and recorded their data prospectively) and the large number of centers and medical specialties that participated in patient recruitment, which provided a large, representative sample of patients with idiopathic TMA and their different clinical aspects.

ADAMTS13 activity has been validated as a crucial criteria for TMA diagnosis [1] and it may also be useful in the future for the guidance of first and second line therapeutic options. However, at short term, the measurement of ADAMTS13 in emergency is unlikely to be available in all hospitals. Therefore, the primary objective of our study was to try to establish a predictive score for severe ADAMTS13 deficiency based on standard biological parameters. The original diagnostic score we established, based on the differences we identified between patients with ADAMTS13 deficiency and those with a detectable ADAMTS13 activity (≥20%) (ie, serum creatinine level, platelet count and, to a lesser extent, ANA positivity) may therefore rapidly identify patients with acquired, immune-mediated ADAMTS13 dysfunction. Of particular interest, creatinine level ≤200 µmol/L and platelet count ≤30×10^9^/L had the stronger association with a severe ADAMTS13 deficiency. By using those 2 criteria, we had a remarkably low number of misclassified patients. Interestingly, we noted that all 3 misclassified patients with a severe renal failure who finally had a deficient ADAMTS13 activity were ≥55-year-old. Since the mean age at TTP diagnosis is usually the fourth decade, this result suggests that older patients may be a risk factor of developing severe renal failure despite ADAMTS13 deficiency. Further studies should thus specifically assess the presentation of patients ≥55-year-old to establish whether age influences clinical presentation in TTP.

Our secondary objective was to focus on idiopathic TMA presentation and short term outcome as a function of ADAMTS13 level (severe or mild deficiency/detectable activity) in order to see if a pathophysiology-based classification could be relevant for therapeutic management. In this work, we failed to associate ADAMTS13 activity with survival. This result contrasts with some previous reports which associated detectable ADAMTS13 activity with an increased death rate [8], [17], [24]–[26]; for review, see [10]). However, these discrepancies may be explained by the fact that we excluded from the analysis TMA associated with other conditions (transplantation, cancer and chemotherapy or HIV infection), which are usually associated with detectable ADAMTS13 activity and a very poor prognosis. Indeed, when only idiopathic TMA are considered, it is likely that ADAMTS13 activity does not distinguish survivors from non survivors.

Importantly, in our study, patients with detectable ADAMTS13 activity had a frequent evolution to end-stage renal disease, similar to patients with HUS [16], [17]. Indeed, idiopathic TMA with detectable ADAMTS13 activity share features of atypical HUS and antiphospholipid syndrome [27] and probably includes various subsets of diseases with distinct pathophysiological mechanisms that require now to be clearly identified. For example, we cannot exclude that a dysfunction in complement proteins may be involved in some of these patients, which may account for the 14% relapse rate we observed. Similarly, the incidence of malignant hypertension or microangiopathic antiphospholipid-associated syndrome, a more recently identified form of TMA [27], remains to be determined in those patients. Last, it is noteworthy that 31 out of 54 patients with a detectable ADAMTS13 activity had a moderate decrease in the protein activity that ranged from 20% to 50%, as it was reported in various autoimmune diseases or other conditions [28], [29]. In these patients, we cannot totally exclude that a subtle dysfunction in ADAMTS13 by yet unknown mechanisms may also have a role in the TMA process.

Surprisingly, we observed no significant difference in term of plasma volume or time to platelet count recovery between the 2 groups of patients. This may be explained by the use of rituximab in 27 patients within the subgroup with a deficient ADAMTS13, though we cannot totally exclude a possible additional role of steroids since patients in the deficiency group received more frequently steroids than patients of the detectable group. Indeed, it is likely that, with only standard plasma-exchange-based treatment, these 27 patients may have required longer treatment duration and may have needed more time to achieve a normal platelet count. However, interestingly, we confirm here that ADAMTS13 activity is of prognostic value since an acquired enzyme deficiency on presentation is associated with more frequent flare up episodes of the disease despite intensive treatment (for review, see [10]). Those exacerbations are associated with an increased duration of hospitalization stay and with an increased therapy-related morbidity [30], and they represent a typical current indication of additional therapies such as rituximab, aimed at reducing the number of plasma exchange sessions and treatment duration [11]–[14].

In conclusion, we provide here an original and reliable diagnostic score involving platelet count and serum creatinine level to predict severe acquired ADAMTS13 deficiency on diagnosis. Of course, this score is not intended to substitute for measuring ADAMTS13 activity, which remains mandatory both to confirm the pathophysiology of the TMA and to validate retrospectively initial therapeutic management. In addition, using a large cohort of patients with idiopathic TMA, we emphasized that a pathophysiology-based classification is likely to be more relevant than a clinical presentation-based classification to predict the short term outcome. Thus, at the very initial step of patient management, this score may be very helpful to identify patients liable to experience exacerbations of the disease during standard treatment and thus potentially requiring early immunomodulatory drugs. This subgroup of specific patients may be a good target for future therapeutic assays goaled to evaluate the accurate timing of immunomodulation in the therapeutic scheme of TMA.

## Supporting Information

Figure S1 Pathophysiological mechanisms leading to microthrombi and organ failure in TTP. The blood smear shows schistocytes (black arrows) as a consequence of thrombi and very high shear stress in microcirculation.
(0.12 MB TIF)
