# Supplementary figures and images for: Predictive Features of Severe Acquired ADAMTS13 Deficiency in Idiopathic Thrombotic Microangiopathies: The French TMA Reference Center Experience
Source: PLoS One. 2010 Apr 23;5(4):e10208. doi: 10.1371/journal.pone.0010208 (PMC2859048; doi:10.1371/journal.pone.0010208)

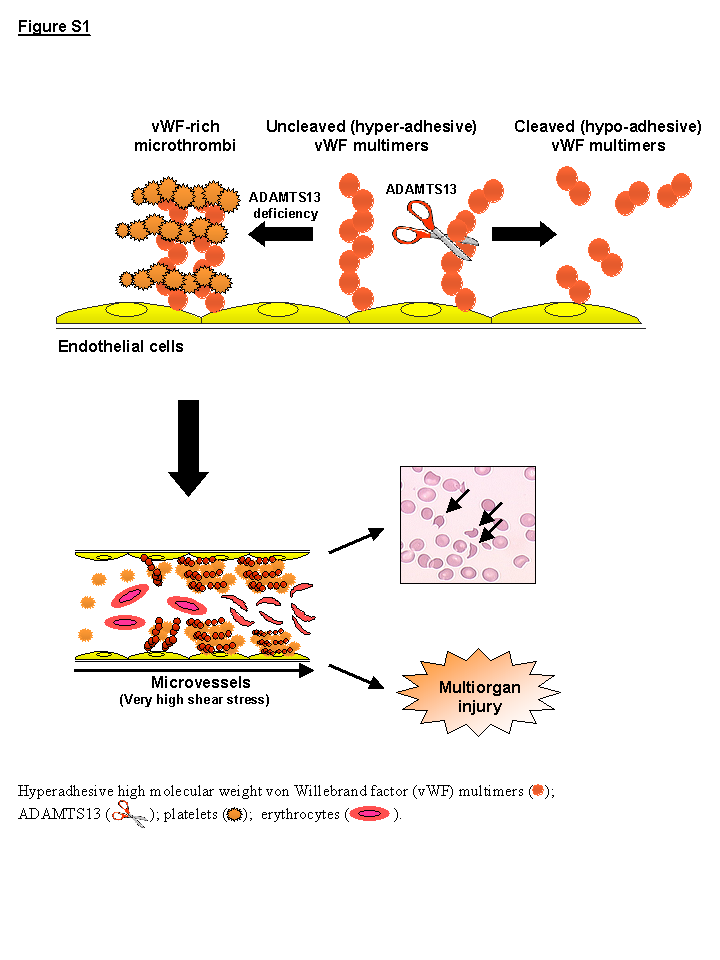

Supplement: Figure S1 — Pathophysiological mechanisms leading to microthrombi and organ failure in TTP. The blood smear shows schistocytes (black arrows) as a consequence of thrombi and very high shear stress in microcirculation. (0.12 MB TIF) [file pone.0010208.s001.tif]
